# Performance in a gaze-cueing task is associated with autistic traits

**DOI:** 10.3934/Neuroscience.2021007

**Published:** 2020-12-17

**Authors:** Mariana FP de Araújo, Wagner A de Castro, Hiroshi Nishimaru, Susumu Urakawa, Taketoshi Ono, Hisao Nishijo

**Affiliations:** 1 System Emotional Science, Graduate School of Medicine, University of Toyama, Toyama, Japan; 2 Department of Physiological Sciences, Health Sciences Center, Federal University of Espirito Santo, Vitoria-ES, Brazil; 3 Department of Musculoskeletal Functional Research and Regeneration, Graduate School of Biomedical and Health Sciences, Hiroshima University, Hiroshima, Japan

**Keywords:** autistic traits, autism-spectrum quotient, cueing tasks, gaze-triggered orienting, feature-based perception, attention switching deficits, over-focused attention

## Abstract

Individuals with autism spectrum disorder (ASD) show impairments in processing social cues such as facial expressions and gaze direction. Several researchers have proposed that autistic traits form a continuum that may be distributed within the general, typically developed, population. Accordingly, several studies have indicated that typically developed individuals with high levels of self-reported autistic traits have autistic-like performance in a variety of paradigms. Here, we designed a gaze-cueing task to examine whether gaze-triggered orienting is related to the extent of typically developed (TD) individuals' autistic traits (determined by their AQ test scores) and whether it is modulated by previous eye contact and different facial expressions. At each trial, TD subjects observed faces with or without eye contact. This facial stimulus then gazed toward the left or right side. Finally, a target appeared on the left or right side of the display and reaction time (RT) to the target was measured. RTs were modulated by congruency between gazing directions and target locations, and by prior eye contact in the congruent trials. In addition, individuals with higher AQ scores were slower at detecting the target when the cue was a happy face. Furthermore, faster RTs in congruent trials were associated with one specific autistic trait (attention switching deficits). Together, these results indicate that autistic traits may influence performance in a gaze cueing task.

## 1. Introduction

Autism spectrum disorder (ASD) is characterized by stereotyped behavior and impairments in using social communication cues, such as gaze direction and facial expressions [1]–[3]. The lack of ability in following eye-gaze in a joint attention situation is a great indicator of autism in early childhood [1]. Accordingly, eye track studies show that both children and adults with ASD fixate less on faces than typically developing individuals [4],[5]. In addition, when looking at faces, people with ASD show reduced preference for the eye, characterized by both more frequent eye movements away from eyes and by avoidance of direct eye contact [6],[7]. These impairments seem to be related to differences in activation of several brain regions involved in visual, social and emotional processing. Studies using electroencephalography and functional imaging techniques, for example, reported that a set of brain regions that are differentially activated during the observation of direct gaze and/or emotional expressions in ASD compared with typically developed (TD) individuals [3],[8].

In laboratory settings, gaze-cueing tasks are commonly used to study attention orienting to the direction of another's gaze [9]. In these tasks, a central face gazing to left or to right is first presented, and then a lateral target appears at the side (congruent trials) or at the opposite side (incongruent trials) of the gaze. Both non-autistic adults and children are faster to detect or identify the target in congruent trials. Such congruency effect, or gaze-cueing effect, is a measurement of our tendency to attend to the location indicated by the gaze of another person. Importantly, such gaze-cueing effect can be modulated by both facial expressions and previous direct eye contact [10],[11]. Most studies in people with ASD also reported a gaze-cueing effect, suggesting that these individuals reflexively orient to gaze (for a review, see [1]). However, studies comparing the congruency effect elicited by gaze cues and by non-social cues (such as arrows) identified differences in gaze-triggered attention orienting between TD and autistic individuals. Specifically, while TD individuals seem to preferentially orient to gaze cues, ASD individuals orient their attention similarly to both non-social and gaze cues (for a review, see [1]). Together, these findings suggest that the mechanisms underlying gaze-triggered orienting in ASD and TD individuals are different, and that in individuals with ASD attention orienting to gaze cues may rely on a more general, non-social, mechanism for symbol direction detection. Specifically, individuals with ASD seem to be sensitive to the physical features of the eyes, such as eye movements or the contrast between the pupil and sclera.

In the past decades, several researchers have suggested that there is a continuum of autistic traits that may be distributed within the general population, with individuals diagnosed with ASD being at the upper extreme of this continuum [12]. According to this view, there is a broader autism phenotype (BAP) in non-clinical population, with some TD individuals having high levels of ASD-related traits [13],[14]. The extent of autistic-like traits, and therefore the point where an individual lies on this autism continuum spectrum, can be assessed by the autism spectrum quotient (AQ) test [12]. Accordingly, a growing number of studies show that TD individuals with high scores in the AQ test have autistic-like performance in a variety of tasks. In this context, a previous study indicated that individuals with lower AQ scores tended to look at direct relative to averted eyes, but individuals with high AQ scores did not [14]. Higher scores in the AQ test were also associated with impaired interpretation of nonverbal aspects of social communication, such as hand gestures and facial expressions [15], impaired facial recognition [16] and impaired social learning [17],[18]. In addition, a few studies also reported differences in gaze-triggered orienting in TD subjects with low and high levels of autistic traits [18]–[21]. However, it is still not fully understood how gaze orienting in individuals with different levels of autistic traits is differentially modulated by facial expressions and direct eye contact. In the present study, we designed a pre-cueing task to further investigate the relationship between autistic traits and gaze-triggered orienting in TD population, and whether it is influenced by the facial expression of the cue stimulus (neutral, happy and anger) and by prior direct eye contact.

## 2. Materials and method

### 2.1. Subjects

Twenty subjects (male: *n* = 8, female: *n* = 12, average age 22.3 ± 2.28) participated in this experiment. All of them were undergraduate students in the University of Toyama and naive to the purpose of the experiment. They were all right-handed and had normal visual acuity with or without correction. All the experimental procedures were performed according to the ethics code of our institution, with adequate understanding and written informed consent of the subjects and were approved by the Ethical Committee of Human Experiments at the University of Toyama. One subject was discarded because she did not participate in the Autism Spectrum Quotient (AQ) test, and another was discarded due to technical problems in data collection.

### 2.2. Experimental setup

#### 2.2.1. Stimuli

**Figure 1.**
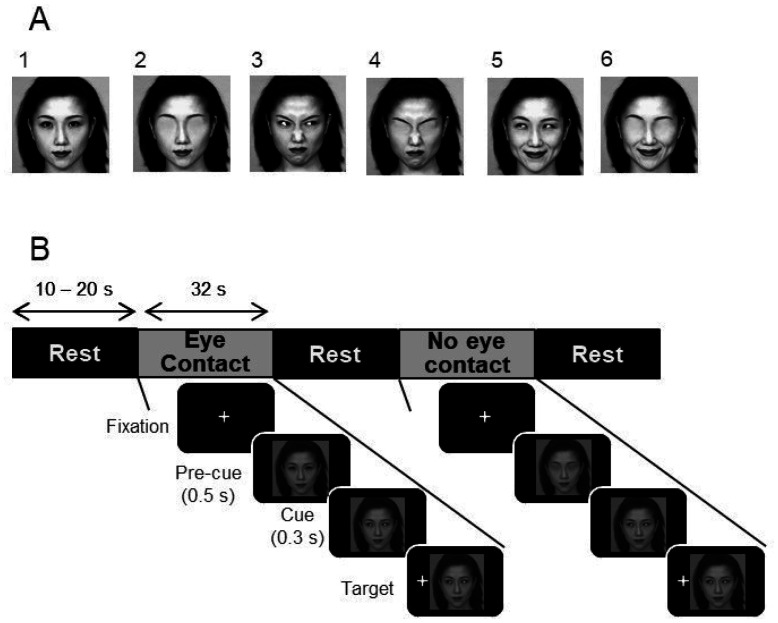
Schematic representation of the task. (A) Six examples of the stimuli used in the task. (1–2), neutral faces gazing towards the observer (1) and without the eye region (2); (3–4), anger faces gazing towards the right side (3) and without the eye region (4); (5–6), happy faces gazing towards the left side (5) and without the eye region (6). (B) Experimental design. One experimental session consisted in alternated eye contact and no eye contact condition task blocks, separated by inter-block intervals of 10–20 s. In each block, the 12 combinations of cue stimuli (3 facial expressions and 2 types of congruency) and target (left and right) were randomly presented. Each trial started with a warning buzzer and a fixation cross displayed at the screen. Then, a pre-cue stimulus appeared, followed by a cue stimulus. Finally, the target (white cross) appeared at the left or right side of the screen. Illustrations are original drawings by M.F.P.A., W.A.C., and H. Nishij. Facial pictures are shown by permission of ATR-Promotions (rights holder).

Facial stimuli consisted of 12 upright face images of a Japanese female model, with different expressions (anger, neutral or happy) and gaze directions [left, right and center (straight gaze to the subject)]. These pictures were selected from the ATR Database, in which the emotional categories were psychologically assessed [22]. Thus, 3 pictures were created for each facial expression, each of them containing one of the possible gaze directions (a total of 9 facial stimuli). Another 3 facial images with different emotions (anger, neutral or happy) but without the eye regions were created by erasing their eye regions from 3 facial expressions with direct gaze using Photoshop software. Examples of the stimuli with and without eyes are shown in Figure 1A. All these photographs were subtended 16° in height and 16° in width of the visual field. The photographs were displayed using 8-bit RGB color levels on a black background. All stimuli were adjusted to reduce the number of distractors.

#### 2.2.2. Apparatus

A personal computer (Power Macintosh 9500/150) and a 17-inch color monitor (FlexScan P1700) were used to present the stimuli using E-prime software (Psychology Software Tools, Inc). The subjects sat in a dark room, and their heads were placed on a chin-rest 60 cm away from the screen. They were instructed to keep the right index finger lightly touching the downward arrow key so that they could easily press the leftward or rightward arrow keys when required, and to keep their left hand resting on their left leg while they watched the computer monitor.

### 2.3. Task procedure

Each trial started with a warning buzzer sound for 0.5 s, followed by a fixation cross appearing in the center of the screen for 1 s. Then, either a pre-cue facial stimulus with a center gaze (eye contact condition) or without the eye region (no eye contact condition) appeared for 0.5 s. A second facial stimulus with the same emotional expression as the first facial stimulus (cue) then appeared for up to 1.3 sec. This face gazed toward the right or left side at 45°. Finally, 0.3 s after onset of the cue stimulus, a white cross (target) appeared either on the left or right side of the screen (14° horizontally away from the center of the screen). Thus, there was a 0.3 s stimulus onset asynchrony (SOA) (onset time difference between cue and target) in this experiment. Figure 1B shows a schematic depiction of the sequence of events in one trial. There were two kinds of trials based on the relation between the gaze direction of cue stimuli and target location. In congruent trials, the direction of the gaze was the same as the target position, and in incongruent trials gaze direction was opposite to the target position.

The subjects had 1 s to locate the target and press the leftward or rightward arrow key according to the location of the target. When the target appeared on the right, the subject was instructed to press the rightward arrow key as quickly as possible, while when the target appeared on the left, the subject had to press the leftward arrow key. If it took more than 1 s for the subject to push the keys, that trial was considered to be unanswered, and automatically terminated. If the subject pressed the key indicating the side opposite to the location of the target, the response was considered to be incorrect. All trials were carried out in the same way except for presence or absence of the eye region in the first facial stimulus, differences in the facial expressions, and congruity between the gaze direction and target position.

The experiment was divided into two phases: training and test. The training period lasted for 3 min, which was equivalent to the period required for around 90 trials. The test session consisted of 9 blocks in the eye contact condition and 9 blocks in the no eye contact condition. These 2 types of blocks alternated, and each block was separated by inter-block intervals of 10–20 s. In each block, the 12 combinations of cue stimuli (3 facial expressions and 2 types of congruency) and target (left and right) were randomly presented.

### 2.4. AQ test

The subjects were asked to fill in the Japanese version of the AQ test [23] one month after they completed the experiment. The AQ test consists of 50 questions and is divided into five different domains (with 10 questions each) associated with the autistic spectrum: social skills, attention to detail, attention switching, communication and imagination. The subjects score 1 point for each “autistic-like” answer. Therefore, the score in each domain ranges from 0 to 10 and the AQ final score is the sum of the scores in the 5 subcategories.

All questionnaires were answered with no time limit and without intermediation of the researcher. The subjects were not informed that the survey quantified the levels of autistic spectrum. After completion of the questionnaire, the total score and the scores for each domain of the AQ test were determined for each subject. Then, the scores were divided into quartiles. Subjects with scores equal or smaller than the lower quartile (n = 6, scores between 9 and 14) were assigned to the low AQ group, those with scores higher than the upper quartile (n = 3, scores between 21 and 26) were assigned to the high AQ group, and those with scores between the lower and upper quartiles (n = 9, scores between 15 and 20) were assigned to the medium AQ group.

### 2.5. Statistical analysis

Incorrect responses and responses faster than 0.1 s or slower than 0.9 s (0.37% of the total) were excluded from the analysis. Averaged raw reaction time (RT) data were combined and averaged for each of the 12 conditions for each subject (2 types of the eye contact conditions × 3 emotional expressions × 2 types of congruency). The raw RT data were also *z*-transformed for each subject to normalize the differences in RTs among the subjects. This normalization is effective to analyze the data when the variation in the raw mean RTs among subjects is high [10]. All data was tested for the assumptions of normality (Shapiro-Wilks test), sphericity (Mauchly's test) and homogeneity of variance (Bartlett's test).

The raw data were compared by a four-way mixed ANOVA with group (low, medium and high AQ) as a between-subject factor and pre-cue condition (eye contact, no eye contact), congruency (congruent, incongruent) and emotional expression (angry, happy, neutral) as within subject factors. The normalized data was compared by a similar four-way mixed ANOVA. Subsequent multiple comparison analyses were performed by Ryan's method and simple main effects. P-values smaller than 0.05 were defined as statistically significant.

Since autistic traits are often considered as a continuous factor, simple linear regression analyses were performed to further assess the potential relationship between autistic traits and performance on the task. Specifically, simple linear regressions were calculated to predict RTs based on AQ scores and on the subdivisions of AQ scores (social skills, attention to detail, attention switching, communication and imagination). The simple linear regressions were calculated separately in the congruent and incongruent trials in the eye contact and no eye contact conditions.

## 3. Results

### 3.1. Raw data analysis

**Figure 2.**
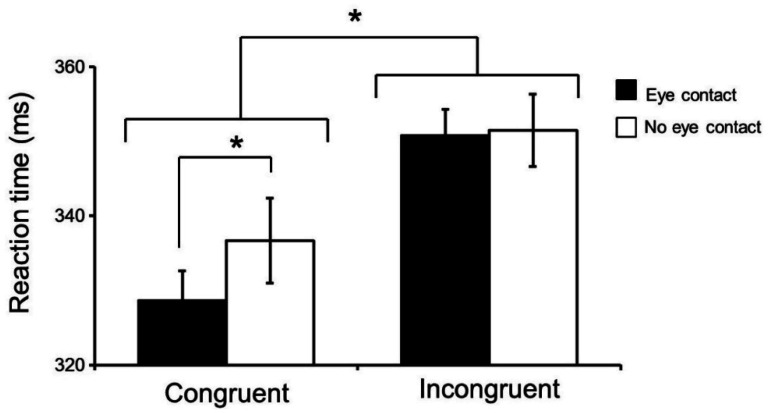
Effects of congruency and eye contact on reaction time (RT). The data indicate averaged RTs (ms) in the congruent and incongruent trials in the eye contact and no eye contact conditions. The data are presented as mean ± S.E.M. * : significant difference at p < 0.05.

The four-way mixed ANOVA analysis of the raw RT data showed a strong main effect of congruency [F (1, 15) = 9.514, p = 0.00076], with higher RTs in the incongruent trials. This four-way ANOVA also indicated an interaction between eye contact and congruency. In addition, there was an interaction between eye contact and congruency [F (1, 15) = 6.353, p = 0.0235]. This interaction is shown in Figure 2; RTs were faster in the eye contact than no eye contact conditions in the congruent trials [F (1, 30) = 7.305, p = 0.012], and the RTs were longer in the incongruent than congruent trials in both the eye contact [F (1, 30) = 13.401, p = 0.001] and no eye contact conditions [F (1, 30) = 5.145, p = 0.0307]. No other significant main effects or interactions were found.

### 3.2. Normalized data analysis

**Figure 3.**
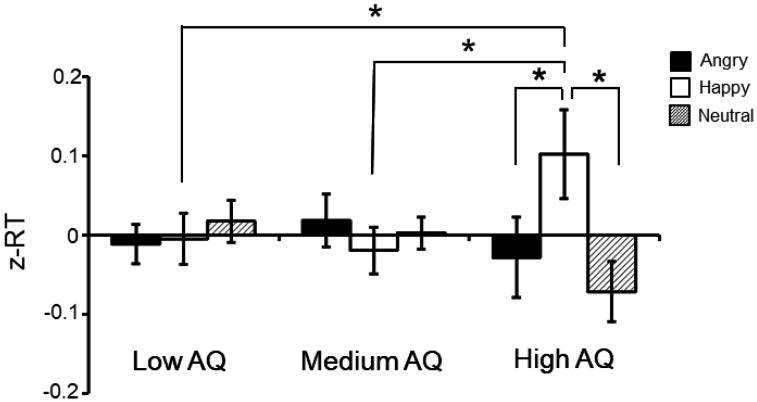
Effects of facial expressions on reaction time (RT) in the three AQ groups. The data indicate mean normalized RTs for each facial expression (anger, happy and neutral) in the low, medium and high AQ groups. The data are presented as mean ± S.E.M. * significant differences at p < 0.05.

Since the tendencies and differences across the groups and conditions might be masked due to differences in RTs between the subjects, the raw RT data were converted to z-score. The four-way mixed ANOVA of the z-scored data indicated a main effect of congruency [F (1, 15) = 8.505 p = 0.016], and an interaction between eye contact and congruency [F (4, 30) = 4.387, p = 0.0367], replicating the results observed in the raw RT analysis. There was also a significant interaction between facial expression and AQ group [F (4, 30) = 4.387, p = 0.0065]. In the high AQ group, the RTs for happy faces were significantly higher than for both neutral [t = 3.345, p = 0.0022] and angry [t = 2.508, p = 0.0177] faces (Figure 3). In addition, the z-score analysis revealed that the high AQ group located the target slower than the medium [t = 3.511, p = 0.0010] and low [t = 2.909, p = 0.0056] AQ groups when the cue was a happy face (Figure 3).

### 3.3. Linear regression analysis

**Figure 4.**
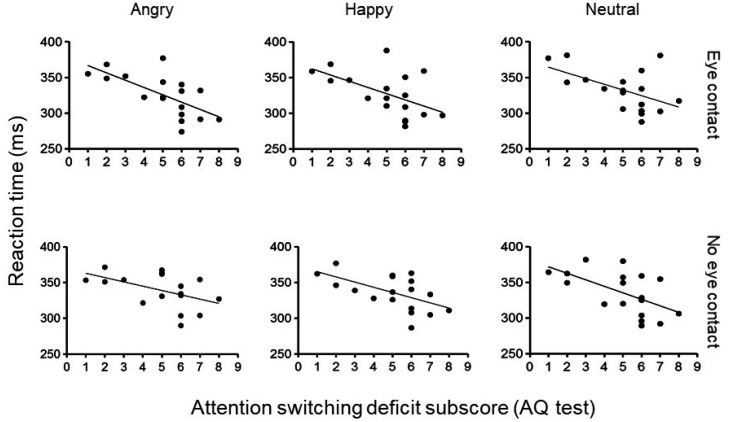
Linear regression analyses between raw reaction time (RT) and attention switching deficit subscores for each facial expression in the congruent trials. Individuals with higher attention switching subscores performed the task faster. Upper: Simple linear regression analyses between RTs and attention switching deficit subscores for angry, happy and neutral faces in the eye contact condition. Lower: Simple linear regression analyses between RTs and attention switching deficit subscores for angry, happy and neutral faces in no eye contact condition.

No significant regression equation was found for total AQ scores. However, significant regression equations were found for attention switching scores in congruent trials (Figure 4). Figure 4 (upper panel) shows the simple linear regression analyses between the RTs and attention switching deficit scores in the congruent trials in the eye contact condition when the angry [F (1, 16) = 12.964, p = 0.002; r^2^ = 0.448], happy [F (1, 16) = 6.641, p = 0.020; r^2^ = 0.293] and neutral [F (1, 16) = 6.192, p = 0.024; r^2^ = 0.279] faces were presented. Figure 4 (lower panel) shows the simple regression analyses in the congruent trials in the no eye contact trials when angry [F (1, 16) = 5.065, p = 0.039; r^2^ = 0.240], happy [F (1, 16) = 7.744, p = 0.013; r^2^ = .326] and neutral [F (1, 16) = 7.827, p = 0.013; r^2^ = 0.329] faces were presented.

## 4. Discussion

### 4.1. Congruency and eye contact effects

This study examined whether gaze-orienting in a pre-cueing task is related to the extent of TD individuals' autistic traits (determined by their AQ test scores) and whether it is modulated by previous eye contact and different facial expressions. The results indicate that all subjects, regardless of their AQ scores, responded faster when the cue face gazed at the same side of the target. These results are consistent with previous findings in TD subjects [10],[24],[25]. Similar cueing effects were also described in individuals with ASD [26]–[28]. Together, these findings suggest that the gaze-triggered attention orienting that underlies the cueing effect is present across the autism spectrum.

In addition, RTs in the congruent trials were faster in the eye contact condition than in the no eye contact condition. Such eye contact effect was observed in all AQ groups. This indicates that the presence of the direct gaze in the pre-cue stimulus directly affected gaze-triggered orienting. Similar influences of prior direct eye contact in both covert and overt gaze-triggered orienting have been previously described in TD individuals [11],[29]. Together, these results indicate that gaze following is influenced by prior eye contact and suggest that there are no differences in the pre-cue eye contact effect among TD individuals, irrespective of their AQ scores.

### 4.2. Differences between AQ groups

The z-scored data analysis indicated that the high AQ group differentially responded to happy faces, since the RTs were significantly higher for happy faces than for both neutral and angry faces. In addition, the high AQ group located the target slower than the medium and low AQ groups when the cue was a happy face. Accordingly, a previous gaze cueing paradigm study found a decreased gaze-cueing effect for happy faces compared to fearful faces in individuals with high AQ scores [20]. In addition, this study found a negative correlation between gaze-cueing effect and AQ scores, suggesting that gaze-triggered orienting to happy faces is weaker in individuals with higher AQ scores compared to individuals with lower AQ scores [20]. These findings may be ascribed to the proposition that individuals with high levels of autistic traits rely more on featural-based processing when performing gaze-cueing tasks. In this context, the happy faces would contain salient features farther from the eye region (such as a wide mouth), which could interfere with task performance in individuals with high AQ scores.

The ANOVA results, however, indicated no measurable effect of AQ score on gaze-triggered orienting, since no interaction between AQ groups and congruency was found. This contrasts with a previous study that reported a negative correlation between AQ scores and gaze-cueing effect, but no correlations between AQ scores and arrow-cueing effect [19], suggesting a reduced salience of social gaze in individuals with high autistic traits. The present results also contrast with another recent study that reported that, in a gaze-cueing paradigm, the cueing effect was larger for individuals with low AQ than for individuals with high AQ [21]. The discrepancies between the present and previous results might be ascribed to the small number of subjects in the current study. Further studies with larger sample sizes are therefore required to confirm the previously reported relationship between autistic traits and gaze-triggered orienting.

Finally, the results indicated that there was a significant negative correlation between attention switching deficits subscores of the AQ test and RTs in the congruent trials for all emotional expressions in both eye contact and no eye contact conditions. Thus, in the congruent trials, the greater the attention switching deficits, the faster the subjects detected the target. It is possible that deficits in attention switching (greater attentional focus) in TD individuals may lead to a greater local attentional bias to the salient features of the stimuli (i.e., eyes), which could explain the shorter RTs in congruent trials found in the present study. Accordingly, a previous study found that infants at-risk of developing ASD tended to sample fewer regions in an array of stimuli, a finding that the authors linked to a possible emergency of an overly focal attention style [30]. An additional possibility is that attention switching deficits may also be related to a dysfunctional arousal regulation by the alerting system. Such dysregulation could result in a tendency to over-focus on the task and a resistance to disengage from the task, which has been previously proposed to be one mechanism underlying the well described superior performance that individuals with ASD show on visual search tasks [31]. Therefore, higher attention switching deficits scores might reflect an over-focused attention on the task, which could, in turn, result in the faster RTs observed in the present study.

## 5. Conclusion

Overall, despite the small sample size, the present results suggest that autistic traits may influence RT in a gaze cueing paradigm. Specifically, individuals with higher AQ scores were slower at detecting the target when the cue was a happy face. In addition, in congruent trials, RTs were negatively correlated with the attention switching subscore of the AQ test. These findings might be ascribed to differences in perceptual and attentional processing amongst TD individuals. Specifically, a more feature-based perceptual processing and an over-focused attention to the task, which are characteristic of ASD, could also extend to the TD population with high levels of autistic traits. Future studies with a larger number of participants, however, are still needed to confirm these findings.
